# 伴EBF1-PDGFRB融合基因阳性急性淋巴细胞白血病2例报告并文献复习

**DOI:** 10.3760/cma.j.issn.0253-2727.2023.01.013

**Published:** 2023-01

**Authors:** 以乔 陈, 湧智 郑, 健 李, 雪玲 华, 少华 乐

**Affiliations:** 福建医科大学附属协和医院小儿血液科，福建省血液病研究所，福建省血液病学重点实验室，福州 350001 Department of Pediatric Hematology, Fujian Medical University Union Hospital, Fujian Institute of Hematology, Fujian Provincial Key Laboratory on Hematology, Fuzhou 350001, China

Ph样急性淋巴细胞白血病（ALL）是2009年首次报道的B-ALL新亚型[1]，其无Ph染色体异常或BCR-ABL融合基因表达，但基因表达谱与Ph阳性ALL高度相似，通常伴有淋巴转录因子基因异常，多预后不良[2]。Ph样ALL遗传学异常主要分为ABL基因异常和JAK-STAT信号通路异常。EBF1-PDGFRB基因融合阳性ALL是较为少见的一种ABL基因异常Ph样ALL，目前国内EBF1-PDGFRB融合基因阳性儿童ALL罕见报道。我们回顾性分析我院进行血液肿瘤全转录组检测的219例ALL患儿，发现仅有2例（0.9％）伴EBF1-PDGFRB融合基因，现对该2例患儿的病例资料报告如下并进行文献复习。

## 病例资料

例1，男，12岁，以“面色苍白伴头晕1个月余”为主诉于2019年12月8日就诊我院。体格检查：神志清楚，精神反应可，轻度贫血外观，呼吸平稳。双颈部触及黄豆大小淋巴结，心肺查体无阳性体征，腹软，无压痛，肝肋缘下2.5 cm，脾肋缘下3 cm。血常规：WBC 248.41×10^9^/L，HGB 93 g/L，PLT 127×10^9^/L，幼稚细胞89％。骨髓象：原始幼稚淋巴细胞80.5％。骨髓免疫分型：92.4％细胞表达CD22、CD34、CD19、CD10、HLA-DR、CyCD79a，为幼稚B细胞。IKZF1突变：外显子2～7杂合缺失。FISH检测：PDGFRB重排，阳性率89％（图1A）。白血病相关融合基因检测（43种）：阴性。染色体核型：46,XY[10]。血液肿瘤全转录组检测：EBF1-PDGFRB融合基因。按CCCG-ALL-2015方案化疗，予地塞米松诱导化疗，化疗第5天外周血幼稚细胞96.15×10^9^/L，予PVDL方案化疗［长春新碱（VCR）1.5 mg/m^2^×4次+柔红霉素（DNR）25 mg/m^2^×2次+培门冬酶（PEG-ASP）2000 U/m^2^×1次］，因合并急性胰腺炎取消第2次PEG-ASP化疗，于诱导化疗第5天开始口服达沙替尼靶向治疗，化疗第19天骨髓象示原始幼稚淋巴细胞77.5％，微小残留病（MRD）88.83％，期间行腰椎穿刺鞘内注射3次，脑脊液检查未见异常。之后行CAT方案（环磷酰胺+阿糖胞苷+巯嘌呤）化疗，化疗第46天复查骨髓象示原始幼稚淋巴细胞1％，MRD 12.07％，IKZF1突变阴性。继而行CAT+VCR+左旋门冬酰胺酶（L-ASP）方案化疗，复查骨髓象示原始幼稚淋巴细胞3％，MRD 6.69％。之后行CAR-T细胞桥接半相合造血干细胞移植治疗，移植后1个月复查骨髓象示缓解，MRD<0.01％，IKZF1突变阴性，EBF1-PDGFRB融合基因阴性，目前无病生存19个月。

例2，男，10岁，以“发现颈部肿物1个月余，面色苍白半个月余”为主诉于2020年1月16日就诊我院。体格检查：神志清楚，精神反应可，重度贫血外观，呼吸平稳。颈部、腋窝、腹股沟触及多发黄豆至花生米大小淋巴结，腹部膨隆，肝肋缘下8 cm，脾重度肿大，Ⅰ线10 cm，Ⅱ线9 cm，Ⅲ线0 cm。血常规：WBC 620.65×10^9^/L，HGB 49 g/L，PLT 26×10^9^/L，幼稚细胞90％。骨髓象：原始幼稚淋巴细胞98.5％。骨髓免疫分型：94.6％细胞表达CD22、CD19、CD10、CD20、HLA-DR、CyCD79a，为幼稚B细胞。IKZF1突变：外显子4～7杂合缺失。FISH检测：PDGFRB重排，阳性率96％（图1B）。白血病相关融合基因检测（43种）阴性。染色体核型：46，XY[20]。血液肿瘤全转录组检测：EBF1-PDGFRB融合基因，PAX5-ZCCHC7融合基因。睾丸彩超：双侧睾丸回声减低伴血供增多（血液病浸润？）。按CCCG-ALL-2015方案化疗，予地塞米松诱导化疗，化疗第5天外周血幼稚细胞244×10^9^/L，予PVDL方案化疗（VCR×3次+DNR×2次+PEG-ASP×1次），于诱导化疗第9天开始口服达沙替尼靶向治疗，诱导期间因合并重症感染及消化道出血取消第4次VCR和第2次PEG-ASP化疗，并停服达沙替尼，化疗第19天骨髓象示原始幼稚淋巴细胞78.5％，MRD 70.16％，期间行腰椎穿刺鞘内注射3次，脑脊液检查未见异常。病情平稳后继续口服达沙替尼，之后行CAT方案化疗，化疗第46天查骨髓象示原始幼稚淋巴细胞3％，MRD 5.22％，FISH检测示PDGFRB重排。继而行CAT+VCR+PEG-ASP方案1个疗程、大剂量甲氨蝶呤（HD-MTX）方案1个疗程、Block3方案（地塞米松+阿糖胞苷+依托泊苷+培门冬酶）1个疗程，复查骨髓象示原始幼稚淋巴细胞14.5％，MRD 32.66％，EBF1-PDGFRB融合基因阳性。于2020年7月行CAR-T细胞治疗，回输后第14天复查骨髓象未见原始幼稚淋巴细胞，MRD<0.01％，回输后第45天复查骨髓象示缓解，MRD 0.11％，EBF1-PDGFRB融合基因阴性。于2020年9月予FABuCy方案预处理化疗，行半相合外周血造血干细胞移植治疗，移植后1个月复查骨髓象示缓解，MRD<0.01％，EBF1-PDGFRB融合基因阴性，PAX5-ZCCHC7融合基因阴性，IKZF1突变阴性，目前无病生存16个月。

**图1:**
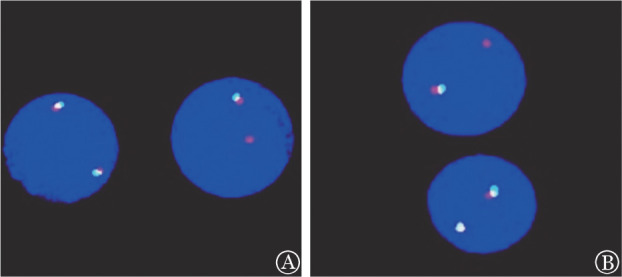
FISH检测显示PDGFRB分离探针为非典型异常信号模式 A 例1； B 例2

## 讨论及文献复习

目前国内外共报道了25例携带EBF1-PDGFRB融合基因的ALL患者（国内3例，国外22例）[3]–[9]，多为青少年（>10岁20例），临床特征为高白细胞血症，FISH检测示PDGFRB重排（21例），诱导缓解率低。Schwab等[6]报道了15例EBF1-PDGFRB融合基因阳性的儿童ALL患者，女性为主（11/15），中位年龄12岁，中位外周血WBC 48.8×10^9^/L。本文报道的2例患者均为青少年，年龄分别为10岁和12岁，初诊WBC>100×10^9^/L，与现有文献报道的临床特点一致。本文2例患者染色体核型正常，FISH检测示PDGFRB重排，经全转录组检测证实EBF1-PDGFRB基因融合。EBF1-PDGFRB融合基因是由染色体5q32-33微缺失引起，染色体核型分析无法辨别、易忽略，而FISH检测用时短，可早期识别该项不良遗传学改变，从而进行治疗方案调整。

PDGFRB重排的ALL多表现为化疗不敏感、MRD水平高、复发率高，应用强化疗联合酪氨酸激酶抑制剂（TKI）治疗可显著提高缓解率[2],[5]–[6],[8]–[9]。Schwab等[6]报道的13例接受早期化疗方案治疗（未使用TKI）的患者中有10例诱导治疗结束时MRD阳性，7例复发（距诊断18～59个月），共9例长期存活。其中3例接受造血干细胞移植治疗，1例移植后复发，均存活。另有2例患者诱导治疗结束MRD>10％，加用伊马替尼后获得持续分子生物学缓解。Roberts等[2]报道了4例伴EBF1-PDGFRB融合基因的ALL患者均诱导化疗失败，加用TKI治疗后MRD短时间内清除、获得持续缓解。另有2例个案报道EBF1-PDGFRB融合基因阳性的难治性B-ALL患者在加用伊马替尼治疗后获得完全缓解[5],[8]。戴海萍等[7]亦报道了1例EBF1-PDGFRB融合基因阳性的ALL患者标准化疗后骨髓MRD持续未转阴，加用伊马替尼靶向治疗并联合CAR-T细胞治疗后处于持续分子生物学水平缓解。Slayton等[10]研究证实了达沙替尼联合化疗的耐受性和安全性良好，同时早期加用达沙替尼联合化疗与伊马替尼联合化疗相比可提高早期缓解率。现有个案报道显示TKI多于诱导化疗未缓解、持续MRD阳性或复发时应用[2],[5]–[6],[8]，而本文2例患者均在明确存在PDGFRB重排时加用达沙替尼治疗。

TKI的应用可改善伴EBF1-PDGFRB融合基因阳性的Ph样 ALL的预后，研究表明造血干细胞移植也可使其获得长期缓解[3],[6]。Roberts等[11]针对儿童ALL的队列研究表明虽然Ph样ALL儿童患者诱导结束时MRD水平高，但基于MRD调整化疗强度的治疗方案（包括造血干细胞移植）使得Ph样ALL患者与非Ph样ALL患者的生存情况无明显差异。本文报道的2例患者虽在诱导期间即加用达沙替尼，但早期治疗反应差，化疗第19天骨髓象呈M3状态、骨髓MRD>10％，化疗第46天骨髓MRD>1％，其中例2移植前形态学复发，2例患者均在接受造血干细胞移植治疗后获得长期缓解。研究表明伴IKZF1改变的Ph样ALL患者预后差于不伴IKZF1改变的Ph样ALL[2]。Slayton等[10]的研究也显示伴IKZF1缺失突变者预后不佳，可作为是否需要行造血干细胞移植的评判指标之一。国内报道的TKI+CAR-T细胞治疗后复发死亡的患者除EBF1-PDGFRB重排外伴BCORL1基因Q426K突变，另1例经诱导化疗达完全缓解、造血干细胞移植治疗后长期存活的患者未携带其他遗传学改变[3]–[4]。本文2例患者除EBF1-PDGFRB融合基因阳性外均伴有IKZF1杂合缺失，例2同时伴有PAX5-ZCCHC7融合基因，考虑TKI联合强化疗治疗反应差与上述遗传学改变有关。对于伴EBF1-PDGFRB融合基因的Ph样ALL患者建议及时加用TKI联合强化疗治疗，MRD持续阳性者尽早行造血干细胞移植。
